# Optimizing Sunscreen Safety: The Impact of TiO_2_ Particle Size on Toxicity and Biocompatibility

**DOI:** 10.3390/nano15120951

**Published:** 2025-06-19

**Authors:** Adriana S. Maddaleno, Clàudia Casellas, Elisabet Teixidó, Laia Guardia-Escote, Maria Pilar Vinardell, Montserrat Mitjans

**Affiliations:** 1 Department of Biochemistry and Physiology, Universitat de Barcelona, 08028 Barcelona, Spain; adrianamaddaleno@ub.edu; 2 Institute of Nanoscience and Nanotechnology, Universitat de Barcelona, 08028 Barcelona, Spain; 3 Department of Pharmacology, Toxicology and Therapeutic Chemistry, Universitat de Barcelona, 08028 Barcelona, Spain; clcasell8@alumnes.ub.edu (C.C.); eteixido1511@ub.edu (E.T.); laia.guardia@ub.edu (L.G.-E.)

**Keywords:** TiO_2_ nanoparticles, hemocompatibility, toxicity

## Abstract

The use of UV filters is a well-established strategy for preventing skin cancer and photoaging. Among inorganic filters, titanium dioxide (TiO_2_) provides excellent protection against both UVA and UVB radiation. Moreover, the use of such inorganic filters at the nano-sized scale has increased their acceptability because it ensures the cosmetically desired transparency in sunscreens that consumers demand. However, concerns remain regarding the potential toxicity of TiO_2_ nanoparticles, and discussion about their use in pharmaceuticals and cosmetics is still in progress. Their increased (bio)reactivity compared to bulk materials may lead to DNA damage. Furthermore, their capacity to cross dermal, respiratory, and gastrointestinal membranes remains a subject of debate. This study is therefore designed to assess and contrast the toxicological characteristics of a pair of commercially available titanium (IV) oxide sunscreens differing in particle size—microscale versus nanoscale. First, the morphology and hydrodynamic diameter of the TiO_2_ nanoparticles were characterized. Then, potential interactions and/or interferences of these nanoparticles with the methods used to evaluate cytotoxic behavior were studied. Finally, the hemocompatibility, cytotoxicity, phototoxicity, and genotoxicity of both micro- and nano-sized TiO_2_ were evaluated using human keratinocytes.

## 1. Introduction

Ultraviolet (UV) radiation exposure is associated with multiple adverse effects on the skin, notably the induction of skin carcinogenesis as well as premature skin aging caused by repeated exposure to the sun. In this sense, the World Health Organization (WHO) specifies that skin cancer is among the most frequent malignant neoplastic diseases [1] and warns that the forecasts of people that will be affected by mid-century are alarming [2]. Robust epidemiological and experimental evidence linking UV exposure to most melanomas supports the use of sunscreens as a key component in primary prevention.

Sunscreens are intricate formulations comprising both active and inactive ingredients, delivered through various vehicles. The photoprotective properties of these products are predominantly conferred by their incorporated UV filters. Titanium oxide (TiO_2_) is a commonly used inorganic UV filter in sunscreen formulations [3] because of its protective range and broad-spectrum protection, encompassing 280 nm to 400 nm wavelengths [4]. Moreover, TiO_2_ is regarded as a safe molecule, being used in higher concentrations than organic UV filters. The use of micro-sized TiO_2_ (0.1–10.0 μm) as an inorganic sunscreen filter often raises aesthetic concerns, particularly due to the white cast it leaves on the skin, which is especially noticeable on darker skin tones. This drawback can be addressed by replacing micro-sized titanium dioxide (TiO_2_) with its nano-sized counterpart. Particle size plays a critical role in the scattering and reflection of UV radiation: bigger particles scatter more UV radiation but also reflect more visible light, resulting in a noticeable white appearance. In contrast, smaller particles (10–20 nm) reflect less visible light and thus appear nearly transparent, compared to the white appearance of larger particles. However, reducing TiO_2_ particle size to the nanoscale diminishes its UVA absorption capacity and shifts its photoprotective range more toward UVB wavelengths [5,6]. Therefore, while nano-TiO_2_ enhances cosmetic appeal by improving transparency, it compromises UVA coverage.

A critical consideration with nanoparticles is their increased surface area-to-volume ratio, which may enhance their (bio)reactivity compared to bulk materials. This characteristic has raised safety concerns regarding their inclusion in cosmetic products. The capacity of nano-sized TiO_2_ to modulate inflammation, cell proliferation, apoptosis, and carcinogenesis by reactive oxygen species (ROS) has been described by different authors [7,8]. Moreover, nano-TiO_2_ can increase the production of ROS when exposed to light because it is a photoreactive molecule [9]. To address this limitation, nanoparticles are frequently functionalized with inorganic coatings such as alumina or silica, which effectively suppress reactive oxygen species (ROS) generation while improving integration with other sunscreen ingredients [10].

A critical concern associated with the ultrafine dimensions of nano-TiO_2_ is their potential to traverse physiological barriers such as the skin, respiratory epithelium, or gastrointestinal tract. Such penetration raises the possibility of systemic distribution and subsequent toxicological effects, thereby representing a potential risk to human health [11,12]. In the case of skin, TiO_2_ nanoparticles have been identified in both the outer layer of the skin and the dermis, suggesting their potential to penetrate beyond the stratum corneum into deeper cutaneous structures [13]. The use of some formulations such as aerosol sprays allows TiO_2_ NPs to enter the body via inhalation and ingestion and induce harmful effects on organic internal systems and organs. In this sense, the toxic effects of TiO_2_ NPs on respiratory, endocrine, and reproductive systems have been reported using animal models [14]. Finally, regarding the potential risks that TiO_2_ nanoparticles may induce in the gastrointestinal tract, their use as a food additive was banned recently [15], and their use in pharmaceuticals and cosmetics is an open debate in the process of being completed [16].

This work compares the toxicological profile of micro- and nano-sized titanium (IV) oxide powders available on the market. First, TiO_2_ nanoparticles were morphologically observed and their hydrodynamic diameter determined in various media. Then, the potential interactions and/or interferences of such NPs with the methods used to evaluate cytotoxic behavior. Finally, the hemocompatibility of titanium (IV) oxide, as well as its cytotoxicity, genotoxicity, and phototoxicity, was assessed using human keratinocytes.

## 2. Materials and Methods

### 2.1. Particles Studied, Reagents, and Culture Media

- Characteristics of the TiO_2_ studied

The characteristics of the two commercial TiO_2_ particles (Sigma-Aldrich, Madrid, Spain) used in the present study are summarized in Table 1. They have a molecular weight of 79.87 g/mol and a density of 4.26 g/mL at 25 °C.

- Reagents and cell culture media

Phosphate-buffered saline at pH 7.4 was prepared according to [17] using extra-pure potassium dihydrogen phosphate, synthesis-grade sodium chloride (Scharlau, Sentmenat, Spain), and disodium hydrogen phosphate anhydrous (Panreac, Castellar del Vallés, Spain).

Other reagents used in the different assays described in the manuscript, including Bovine Serum Albumin, fibrinogen, dimethyl sulfoxide (DMSO), Thiazolyl Blue Tetrazolium Bromide (MTT), Neutral Red Solution (NR), Trypan Blue Solution, ethanol, and acetic acid, agarose type 1, low-melting-point agarose, N-Lauroylsarcosine sodium salt, NaCl, EDTA, Tris, NaOH, Triton X-100, and 4′,6-diamidino-2-phenylindole (DAPI), were supplied by Sigma-Aldrich (Madrid, Spain). Dulbecco’s Modified Eagle Medium (DMEM) with 4.5 g/L glucose, trypsin protease solution (trypsin/EDTA 0.05%), and sterile phosphate-buffered saline solution (PBS) were purchased from Cytiva (Marlborough, MA, USA). L-glutamine 2 mM and penicillin–streptomycin solution (10,000 U/mL penicillin and 10 mg/mL streptomycin) were obtained from Corning (Glendale, AZ, USA), and 10% Hyclone Fetal Bovine Serum (FBS) was supplied by BioLab (Barcelona, Spain).

### 2.2. Characterization of TiO_2_ Nanoparticles

#### 2.2.1. Transmission Electron Microscopy (TEM)

From a suspension of TiO_2_ particles in water at a final concentration of 10 μg/mL, a drop was deposited on a holey carbon–copper grid and left to dry in the air. Finally, employing a JEOL JEM-2100f microscope (JEOL Ltd., Akishima, Japan), images were obtained to analyze the particle size of the two commercial TiO_2_ powders.

#### 2.2.2. X-Ray Diffraction (XRD) Analysis

X-ray diffraction (XRD) analysis was performed to identify the crystalline phases present in both titanium dioxide (TiO_2_) powders and to assess their crystalline phases.

XRD analysis was carried out using a PANalytical Empyrean Alpha1 powder diffractometer (Malvern Instruments, Worcestershire, UK) operating in Bragg–Brentano θ/2θ geometry with a goniometer radius of 240 mm. A Johansson-type Ge (111) monochromator was used to produce a highly monochromatic incident beam. The X-ray tube was operated at 45 kV and 40 mA. A beam mask limited the axial length of the beam on the sample to 12 mm. Soller slits with an angular width of 0.04 radians were used on both the incident and diffracted beam sides to reduce axial divergence, and a fixed divergence slit of 0.05° controlled the beam width.

Data were collected using a 1D detector (1DER) (Malvern Instruments, Worcestershire, UK) with an active length of 2.122°. Scans were performed over a 2θ range of 4° to 100°, with a step size of 0.026° and a measurement time of 150 s per step. Each measurement was repeated three times to ensure data reliability.

Qualitative phase analysis was performed using the Powder Diffraction File (PDF) database provided by the International Centre for Diffraction Data (ICDD-JCPDS, 2025). Phase identification was based on matching experimental diffraction patterns with standard reference patterns. Rietveld full-profile refinement was employed to conduct a semi-quantitative analysis of the identified crystalline phases. This method also enabled the determination of microstructural parameters, including the average crystallite size.

TEM and XRD were carried out at “Centres Científics i Tecnològics de la Universitat de Barcelona” (CCiTUB).

#### 2.2.3. Dynamic Light Scattering: Diameter and Zeta Potential

Following the protocol described in a previous work [17], the mean hydrodynamic diameter (HD) and polydispersity index (PDI) of TiO_2_ nanoparticles in different media were determined using dynamic light scattering (DLS) with a Malvern Zetasizer ZS (Malvern Instruments, Worcestershire, UK) at a scattering angle of 173° and a refractive index of 2.614, specific to TiO_2_. The nanoparticles (1.0 mg/mL) were incubated in phosphate-buffered saline (PBS, pH 7.4), PBS with albumin or fibrinogen (2 mg/mL), or cell culture media used in the cytotoxicity studies (DMEM 5% FBS, see Section 2.4) for 2 and 24 h at 37 °C. Measurements were taken using a 10 × 10 mm quartz cuvette (Hellma Analytics, Müllheim, Germany) in three sets of five readings.

With the same instrument, we measured the zeta potential of TiO_2_ powders suspended in distilled water at a concentration of 0.1 mg/mL to ensure a uniform suspension with a concentration low enough to minimize multiple scattering effects. All measurements were conducted at 25 °C using disposable folded capillary cells. Each sample was measured almost three times, and the average zeta potential value and standard deviations were reported to assess colloidal stability. Measurements of particles suspended in PBS (pH 7.4) and DMEM 5% FBS were also conducted for comparison purposes.

### 2.3. Hemocompatibility Studies

#### 2.3.1. Red Blood Cell Suspension and Plasma Obtainment

Whole blood samples were freshly obtained via venipuncture from physically fit non-smokers, who abstained from alcohol consumption and were not undergoing any form of pharmacological treatment at the time of donation. Blood collection was performed using tubes containing EDTA or sodium citrate as anticoagulants. All procedures were conducted following the acquisition of informed consent and approval from the Bioethics Committee at the University of Barcelona, Spain (15 February 2021).

To prepare red blood cell (RBC) suspensions, erythrocytes were washed almost three times by adding PBS (pH 7.4) and centrifuging the solution for 10 min (3000 rpm). After each centrifugation, the supernatant was discarded until a transparent and colorless supernatant was obtained. The final RBC suspension was adjusted by adding an adequate volume of PBS to obtain a maximal hemoglobin absorbance at 575 nm of 1.8–2.1. Finally, an appropriate amount of PBS was added to obtain the working RBC suspension as specified in [17].

Plasma for coagulation studies was collected from the blood samples extracted in the presence of sodium citrate and after centrifugation for 10 min at 3000 rpm.

#### 2.3.2. Hemolytic Activity

The capacity of TiO_2_ particles to induce erythrocyte lysis was evaluated by incubating 1.0, 0.5, and 0.25 mg/mL of the particles with 25 μL of RBC in PBS at room temperature for 24 h, as reported previously [17], in dark conditions. The hemolytic capacity of the particles was then evaluated by the absorption of hemoglobin released in the supernatant at 540 nm (Shimadzu UV-Vis 160, Izasa Scientific, L’Hospitalet de Llobregat, Barcelona, Spain) after rapid centrifugation (10,000 rpm, 5 min, Nahita Blue, high-speed centrifuge 2624/2, Sudelab S.L., Rubí, Barcelona, Spain). All experiments were performed in triplicate and independently repeated at least three times. Appropriate controls were included: red blood cells (RBCs) incubated in phosphate-buffered saline (PBS, pH 7.4) served as the negative control (basal hemolysis), while RBCs incubated in deionized water were used as the positive control (complete hemolysis).

The percentage of RBC hemolysis was calculated using Formula (1):

Hemolysis % = [A_Sample_ − A_0%_]/[A_100%_ − A_0%_] × 100, (1)

where *A* is the absorbance obtained for each sample or control, 0% refers to negative control (basal hemolysis) and 100% denotes the positive control or total hemolysis in hypotonic water.

Before studying the hemolytic activity of TiO_2_ particles, potential interferences and interactions of the particles with measurements and reagents assay were discarded.

#### 2.3.3. Prothrombin and Partial Thromboplastin Time Determination

Fresh human plasma was incubated for 30 min at 37 °C under soft rotation in the absence (PBS, pH 7.4) and in the presence of different concentrations (0.1, 0.5, and 1.0 mg/mL) of TiO_2_ particles. Then, in a similar way as previously described [17], we studied how TiO_2_ particles modify the coagulation process by determining the prothrombin time (PT, RecombiPlasTin 2G, HemosIL kit, Werfen, L’Hospitalet de Llobregat, Barcelona, Spain) and the activated partial thromboplastin time (aPTT, SynthASil, HemosIL kit, Werfen, L’Hospitalet de Llobregat, Barcelona, Spain) as a measure of extrinsic and intrinsic coagulation pathways, respectively [18].

Experiments were repeated a minimum of three times using plasma derived from distinct donor sources.

### 2.4. Cell Culture and Cytotoxicity Studies

The human keratinocyte cell line HaCaT (Eucellbank, Celltec UB, Universitat de Barcelona, Barcelona, Spain) was grown and maintained in Dulbecco’s Modified Eagle Medium (DMEM) with 4.5 g/L glucose supplemented with 10% (*v*/*v*) Fetal Bovine Serum (FBS), 2 mM L-glutamine, 100 U/mL penicillin, and 100 µg/mL streptomycin in an incubator at 37 °C, 5% CO_2_. Cell culture media and supplements were acquired at Cytiva (Marlborough, MA, USA), Corning (Glendale, AZ, USA) and BioLab (Barcelona, Spain).

Once cell cultures reached approximately 80% confluence, the medium was removed, and cells were detached using a trypsin solution. The resulting cell suspension was evaluated for viability and density using Trypan Blue exclusion. Cell density was adjusted to 1 × 10^5^ cells/mL, and cells were seeded into 96-well plates. Following a 24 h incubation at 37 °C, in a humidified atmosphere containing 5% CO_2_, cells were exposed for an additional 24 h to varying concentrations of titanium dioxide (TiO_2_) particles. Concentrations of TiO_2_ were prepared from a freshly made stock solution (1.0 mg/mL) in DMEM supplemented with 5% Fetal Bovine Serum (FBS), 2 mM L-glutamine, and 1% antibiotic. Prior to treatment, the culture medium was aspirated, and cells were incubated with serial dilutions of the TiO_2_ suspension for another 24 h. Untreated cells in DMEM were included as viability controls. After exposure, cytotoxic effects were evaluated using three complementary assays: the lactate dehydrogenase (LDH) release assay, 3-(4,5-dimethylthiazol-2-yl)-2,5-diphenyltetrazolium bromide (MTT) reduction assay, and Neutral Red uptake (NRU) assay.

To avoid surface nanoparticle adsorption, LDH was detected in cell-free supernatant without previous centrifugation [19]. Thus, the supernatant of each sample was added to a clear plate and mixed with the same volume of the reaction mixture as specified by the supplier of the cytotoxicity detection kit (Roche 11644793001, Sigma-Aldrich, Madrid, Spain). Incubation was performed at room temperature in dark conditions for up to 15 min, and finally, absorbance at 492 nm (reference wavelength of 600 nm) was obtained by a Tecan Sunrise microplate reader (Männedorf, Switzerland). In each experiment, controls of spontaneous cytotoxicity (untreated cells) and high cytotoxicity (1% Triton-X 100-treated cells) were included. Cytotoxicity was calculated according to the supplier’s instructions following Formula (2):

Cytotoxicity (%) = [([A_EV_ − A_LC_]/[A_HC_ − A_LC_]) × 100] (2)

where *A_EV_* is the absorbance of treated cells, *A_LC_* corresponds to low cytotoxicity values of untreated cells, and *A_HC_* is the absorbance obtained for Triton-X 100-treated cells or high control cytotoxicity. Background absorbance (medium without cells) was subtracted for all samples before calculating cytotoxicity. Then, cell viability was calculated using Formula (3):

Cell viability (%) = 100 − Cytotoxicty (%)(3)

For the MTT and NR uptake assays, we followed the protocols previously outlined by our research group [17]. After removing the supernatant, solutions of the colorants were added to the cells following incubation for at least 3 h in the same conditions as described in previous paragraphs. Crystals formed by the reduction of tetrazolium salts were dissolved in dimethyl sulfoxide (DMSO), whereas dissolution of the NR taken up by viable cells was performed in destain solution containing ethanol and acetic acid. After shaking the plate for 10 min (100 rpm) to ensure even distribution of the contents, the plate was read in a microplate reader at 550 nm (Tecan Sunrise, Männedorf, Switzerland). The percentage of cell survival was calculated, considering that maximal cell viability is afforded by DMEM-treated cells (Formula (4)).

Cell viability (%) = [A_TC_/A_UC_] × 100] (4)

where *A_TC_* is the absorbance of treated cells, and *A_UC_* corresponds to the absorbance of untreated cells.

### 2.5. Phototoxic Behavior

The potential capacity of TiO_2_ to induce phototoxicity was studied in HaCaT cells following the official guideline OECD 432 [20], with slight adaptations.

As detailed in the previous section, cells need 24 h of incubation in complete cell culture media before being exposed to chemicals and forming a monolayer. After that, micro- or nano-sized TiO_2_ (1.95–250 µg/mL) diluted in sterile PBS was applied to HaCaT cells for one hour before cells were concomitantly exposed to light. For each experiment and condition, two plates were prepared: one was exposed to 4 J/cm^2^ of UVA and the other maintained in dark conditions for comparative purposes. At the end of irradiation, supernatants were discarded and replaced by fresh complete medium, and plates were incubated overnight (37 °C and 5% CO_2_). Cytotoxicity was determined by MTT and NRU assays as described above. Each condition was assayed in triplicate, and cells not treated with TiO_2_ were included in all plates.

A photo-irritation factor (PIF) was calculated for each method by the following Formula (5):

PIF = [IC_50_ (−Irr)/IC_50_ (+Irr)] (5)

where *IC*_50_ is the concentration of the test chemical by which the cell viability is reduced by 50% in dark or non-irradiated conditions (−Irr) and in UV-exposed or irradiated conditions (+Irr).

A PIF value greater than 5 indicates phototoxicity, a value between 2 and 5 suggests probable phototoxicity, and a value below 2 indicates non-phototoxicity.

Irradiation was carried out as described previously [21] using Actinic BL 15W/10 FAM/10X25BOX lamps (Philips^®^, 15 W, Amsterdam, The Netherlands) emitting in the UVA region with a peak at ~365 nm. Lamps were systematically checked before irradiation [21]. The irradiation dose was calculated by Formula (6):

E(J/cm^2^) = t(s) × P(W/cm^2^) (6)

where *E* stands for ultraviolet dose, *t* represents the time expressed in seconds and, finally, *P* is the lamp potency.

### 2.6. Genotoxicity

The procedure described in [22] with some modifications was used [17]. Cells were treated with micro- or nano-sized TiO_2_ at three non-cytotoxic concentrations (IC < 30%) calculated from the cytotoxicity studies.

The comet assay, also referred to as single-cell gel electrophoresis, is a sensitive technique for identifying DNA damage in individual cells. In this assay, cells are embedded in agarose gel on a microscope slide, lysed to remove membranes and proteins, and then subjected to electrophoresis. When an electric field is applied, fragmented or damaged DNA migrates away from the nucleus, creating a tail-like pattern resembling a comet, whereas intact DNA stays concentrated in the head region. The length and brightness of the tail correlate with the extent of DNA damage, making this technique useful for assessing genotoxicity and repair processes at the single-cell level.

After the exposure period, the cells were rinsed with 2xPBS and detached using trypsin/EDTA (0.05%, 6 min, 37 °C). Then, cells were mixed at 1:2 with 0.9% low-melting-point (LMP) agarose at 37 °C and spread onto slides that had already been coated with 1% normal-melting-point agarose using a format of 8 mini gels per slide, with 2 gels per concentration. Following overnight incubation at 4 °C in fresh lysis solution containing 2.5 M NaCl, 0.1 M Na_2_EDTA, 10 mM Tris, 1% Triton X-100, and 1% lauryl sarcosine (pH 10), the slides underwent unwinding in an electrophoresis tank by immersing them in cold alkaline electrophoresis buffer (300 mM NaOH, 1 mM Na_2_EDTA) for 40 min at 4 °C. Afterwards, electrophoresis was conducted for 30 min at 300 mA and 0.8 V/cm. Slides were then washed with a neutralizing solution (1 M Tris, pH 7.5) three times and dried. Before the observation of the comets, slides were hydrated by submerging them in Milli-Q water for 10 min. DNA was stained with 20 μL of DAPI solution (5 μg/mL), as this compound binds to the adenine–thymine regions of DNA, acting as a fluorochrome. This compound has a maximum absorption wavelength around 340 nm (UV) and a maximum emitting wavelength of 448 nm (blue) [23].

Under a Nikon TS100 epifluorescent microscope, comets were analyzed using the semi-automated image analysis system Comet Assay IV (Perspective Instruments, Instem, Stone, Staffordshire, UK). The DNA damage was quantified as the percentage of DNA intensity in the tail and tail moment. Three independent experiments (*n* = 3) were performed with duplicates, and 60 randomly selected comets were analyzed in each sample. The five extreme values for tail intensity and tail moment were discarded to ensure a valid representation of the DNA damage, in doing so reducing the high cell-to-cell variability in each slide. As a positive control for genotoxic damage, cells were treated with 250 µM methyl methanesulfonate (MMS) for 24 h.

### 2.7. Statistical Analysis

Experimental replication was established at a minimum of three with triplicates for each condition. Then, results were expressed as mean ± standard error of the mean. A two-way analysis of variance (ANOVA) followed by a two-tailed Student’s *t*-test, Bonferroni test, or Dunnett post hoc test for multiple comparisons was used to determine the differences between the datasets, using the SPSS^®^ program V 27 (SPSS Inc., Chicago, IL, USA). Cases where one-way ANOVA was performed are indicated in figure captions. Differences were considered significant for *p* < 0.05 or *p* < 0.01, as indicated in the figure or table footnotes.

## 3. Results

### 3.1. Transmission Electron Microscopy (TEM) and X-Ray Diffraction Analysis

**Figure 1:**
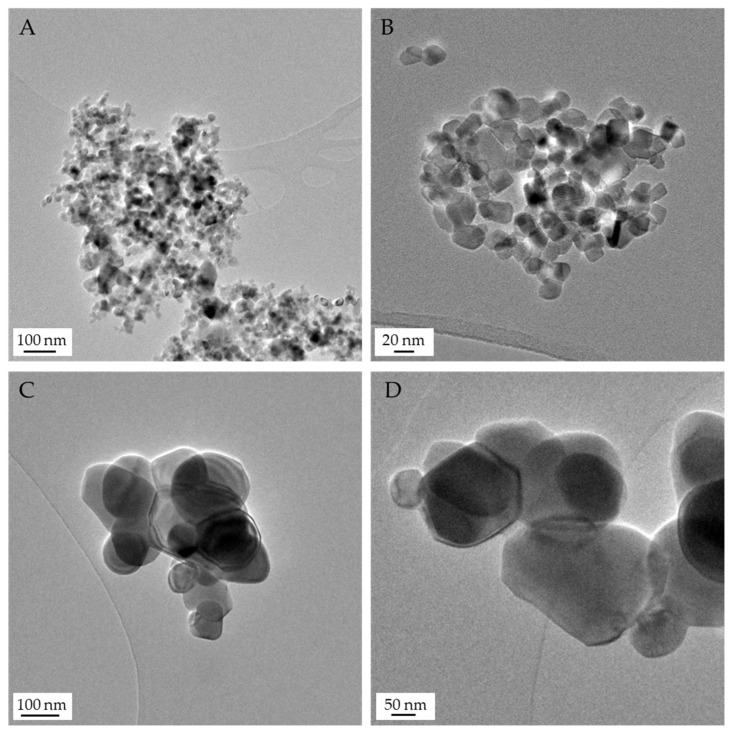
Image of commercial TiO_2_ particles in distilled water when observed with TEM. A TiO_2_ suspension of 10 µg/mL was placed in a grid for observation and the obtention of micrographs with the help of a JEOL JEM LaB6-2100f microscope. (**A**,**B**) 21 nm TiO_2_; (**C**,**D**) micro-sized TiO_2_. Scale bars show micrograph magnification.

Observation of TiO_2_ particles with TEM (Figure 1) indicates that particles tend to agglomerate independently of their size (Figure 1). Morphologically, 21 nm TiO_2_ is round and rod-shaped, resembling the nanoparticles described by other authors [24,25], while micro-sized TiO_2_ particles are mostly round-shaped. Finally, we measured particle diameter and found that nano-TiO_2_ is about 22 nm, corroborating the specifications provided by the commercial supplier, whereas for micro-sized particles, the diameter is approximately 178 nm (see Figure S1).

XRD analysis revealed the presence of crystalline phases in both TiO_2_ powder samples. The identified phases, relative weight percentages, and calculated crystallite sizes are summarized below (Table 2). We found that 21 nm TiO_2_ exhibited a biphasic composition consisting of anatase and rutile (Figure S2A), with an estimated phase ratio of more than 85% anatase and less than 15% rutile, slightly different than the percentages claimed by the supplier (Table 1) and similar to other studies [25]. In contrast, micro-sized TiO_2_ displayed a purely anatase phase (Figure S2B), with no detectable rutile content under the applied measurement conditions. The particle size obtained by XRD was very similar to that measured with TEM in the case of TiO_2_ NPs (considering the percentages of each phase and the crystal size of approximately 20 nm); it was lower in the case of micro-sized powder but in the same range.

### 3.2. Studies of Dynamic Light Scattering (DLS) and Zeta Potential

#### Hydrodynamic Diameter of TiO_2_ and Protein Influence

The high hydrodynamic diameter values obtained for both nano- and micro-sized TiO_2_ particles in the different media studied indicate the existence of agglomerates, corroborating our TEM observations and observations of other researchers [26,27]. This particle agglomeration is not affected by time in the case of 21 nm TiO_2_, but it is affected in microparticles suspended in PBS and PBS with BSA (Table 3). Systems have been described in the literature where stability is apparently reached after 1 h of incubation [28], corroborating this observation in the case of this oxide.

Similarly to our previous studies with ZnO [17], the diameters obtained in the absence of proteins, regardless of the incubation time, are slightly higher than in their presence; although statistical differences were not recorded in all cases, some differences were observed. Moreover, comparing our DLS values at 24 h for 21 nm TIO_2_ (288.2 nm) with those previously reported with free FBS DMEM (472.0 nm) [26] corroborates this hypothesis. The possible justification for this fact is that the addition of proteins can prevent the agglomeration of particles [29] and that protein presence has a stabilizing effect related not to electrostatic repulsion but to steric repulsion [30].

It could be said that the behaviors of the nanometric and micrometric sizes have quite a lot of similarities. The only noteworthy difference to consider is the stability of the nanoparticles in the environment, since the 21 nm NPs have a smaller diameter in DMEM, while micrometric TiO_2_ in the presence of BSA is in the particle size range (123–178 nm).

Another variable analyzed here was polydispersity (PdI). Our samples hold a PdI ranging from 0.4 to 0.6, thus indicating that suspensions have a high PdI and are therefore quite heterogeneous [31] and that an accurate determination of the hydrodynamic diameter is not feasible for these particles.

Altogether, using hydrodynamic diameter and PdI, the behavior of the TiO_2_ particles under various conditions was estimated, in particular, their tendency to cluster and the potential influence of medium components, such as proteins, on their aggregation [29,32]. This approach is commonly used in nanoparticle and colloidal studies to qualitatively assess aggregation or agglomeration behavior in biologically relevant environments, like PBS and culture media [33].

The conclusion we can draw from this test is that, in general, for our TiO_2_ samples, proteins act as deagglomerating agents.

Regarding zeta potential, the initial value obtained for 21 nm TiO_2_ when suspended in distilled water was measured to be approximately 26 mV, indicating moderate colloidal stability (see Supplementary Material Figure S3). However, posterior measures suggest that the particles are being agglomerated or sedimented, making electrophoretic mobility undetectable, corroborating the observations made by TEM and data gathered by DLS. When the nanoparticles were suspended in PBS or DMEM with 5% FBS, zeta potential could not be reliably determined, suggesting a near-neutral surface charge in these media. No detectable zeta potential was recorded for the micro-sized TiO_2_ in distilled water (Figure S4) or in the physiological media. These results can be attributed to particle clustering or low electrophoretic mobility; thus, the behavior of TiO_2_ particles in biologically relevant fluids is significantly compromised by agglomeration or sedimentation [25], but this is the case when TiO_2_ is applied to cells.

### 3.3. Hemocompatibility of TiO_2_

#### 3.3.1. Hemolytic Activity of TiO_2_

**Figure 2:**
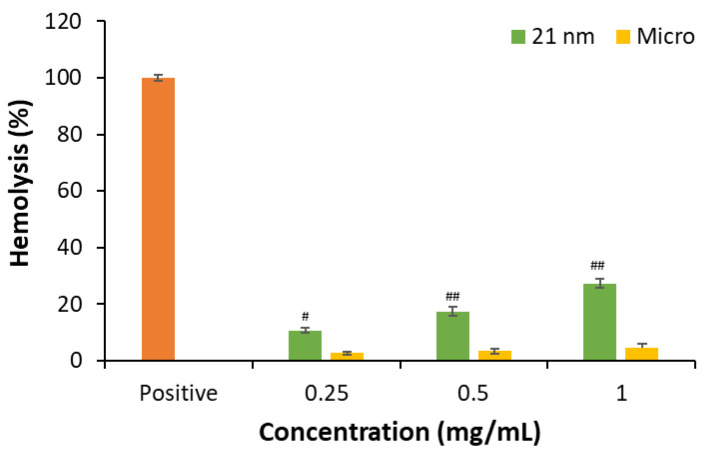
Hemolytic activity of TiO_2_ nano- and microparticles after 24 h of incubation at room temperature and in dark conditions. Results are expressed as the mean ± SEM of three independent assays. The positive control consisted of red blood cells incubated with distilled water under the same conditions as samples with the metal oxide. Statistical differences were assessed by a one-way ANOVA followed by a Student’s *t*-test to assess the effect of particle size (^#^ *p* < 0.05 and ^##^ *p* < 0.01).

We studied the hemolytic capacity of 0.25, 0.50, and 1 mg/mL nano- and micro-sized TiO_2_ particles (Figure 2) after being in contact with an RBC suspension for 24 h at room temperature in the dark. Hemolysis provoked by titanium oxide was determined by spectrometric reading of free hemoglobin.

In the case of micro-sized TiO_2_, no hemolytic effect is observed, while for nano-sized particles, the maximal hemolysis attained is less than 30% (Figure S5). Findings reported by other authors indicate that nanoparticles generally have higher specific surface areas compared to microparticles of the same material [5]. Regarding this, it is assumed that a larger contact surface area in the case of nanoparticles would facilitate greater contact with the erythrocyte membrane, explaining the slight differences in hemolysis observed here for both types of particles [34,35]. However, other characteristics should be regarded because studies with 4–8 nm TiO_2_ nanoparticles showed a non-hemolytic effect [36]. The relationship between hemolytic capacity and zeta potential also presents contradictory aspects, as both positively and negatively charged NPs can induce lysis of the erythrocyte plasma membrane. Moreover, the hemolytic potential of nanoparticles may also be influenced by the source and nature of the erythrocytes and the existence of physical forces [35]. But, in our case, one important factor is the poor colloidal stability when particles, at nano- and micro-sized scales, are suspended in PBS. Our DLS data confirmed that both particle types exist as agglomerates in suspension, and this can reduce their effective surface area and, thus, limit direct interaction with cell membranes in both cases. Therefore, while the assumption of greater surface contact by nanoparticles is supported by indirect evidence and literature data, we recognize that it remains a hypothesis that could not be directly verified in this study.

#### 3.3.2. Effect of TiO_2_ on Prothrombin and Activated Partial Thromboplastin Time

**Figure 3:**
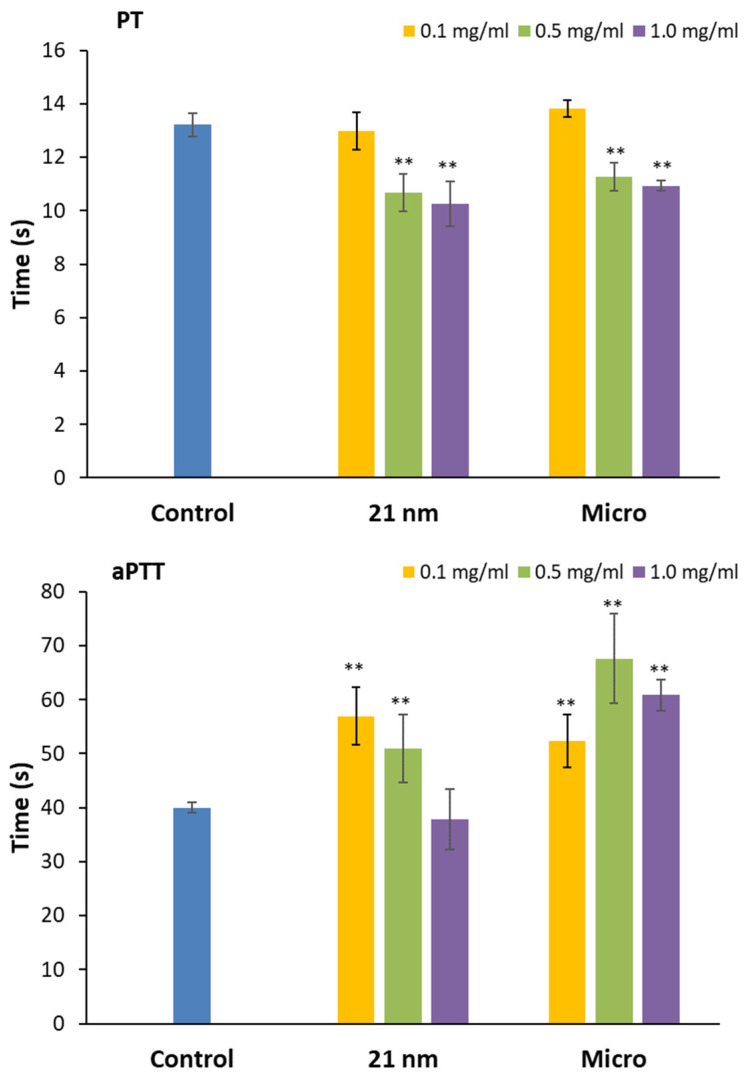
Influence of TiO_2_ 21 nm nanoparticles and microparticles on extrinsic and intrinsic coagulation pathways measured as prothrombin time (PT) and activated partial thromboplastin time (aPTT). Results are expressed as the mean ± SEM of three independent assays. Statistical differences in coagulation time with respect to the control were assessed by a two-way ANOVA followed by a Bonferroni post hoc test. Differences are shown by * *p* < 0.05 and ** *p* < 0.01.

Our data shows that in the case of PT, independently of the size of the particles, there is a decrease in coagulation time with increasing concentrations of TiO_2_ (Figure 3, *p* < 0.01), favoring clot formation [37]. On the contrary, TiO_2_ behaves differently in the case of aPTT depending on the particle size. NPs induce an increase in clotting time, which is inversely proportional to concentration, showing the highest value at the lowest concentration. The presence of micrometric TiO_2_ increases coagulation time with increasing concentrations. For this reason, it was not possible to establish a relationship between particle size and coagulation time. However, regardless of the trend, it can be said that the influence of TiO_2_ on coagulation time is dose-dependent [17,38].

Each nanoparticle has unique physicochemical and morphological characteristics, and its interactions with different components of the coagulation system can vary, even among nanomaterials within the same category [39]. For example, authors have reported that Ag NPs mostly affect aPTT time [40], while in the case of ZnO, both pathways are affected depending on the size of the NPs and concentration [17]. In the case of TiO_2_, [36] described a dose-dependent delay in clot formation in the case of aPTT, in contrast to our findings, but a decrease in PT, like our results, which was independent of the concentration assayed. This different behavior can account for particle size (4–8 nm vs. 21 nm), the presence of rutile crystals included in our sample (20%), or the use of different vehicles instead of PBS. A lower hydrodynamic diameter has been reported for TiO_2_ NPs in RPMI 1640 medium (43%) than the value reported here in PBS, thus indicating less agglomeration of particles that could interact with coagulation factors.

The fact that nano-sized TiO_2_ affects extrinsic and intrinsic coagulation pathways dissimilarly indicates that further investigation should analyze which protein or factor is compromised in each specific coagulation pathway [41].

### 3.4. Cytotoxicity and Phototoxicity of TiO_2_ Particles

#### 3.4.1. Study of Potential Interactions or Interferences with Cytotoxic Assays

The potential interferences of TiO_2_ with reagents used in MTT, NRU, and LDH methods as well as potential interferences in readings have been investigated under cell-free conditions using concentrations of particles ranging from 500 to almost 2 μg/mL. Special attention has been given to potential MTT reduction to formazan or absorption of NR.

Our preliminary observation indicated an increase in absorbance for MTT for both nano- and micro-TiO_2_ independently of the concentration assessed (Figure S6A); however, this low increase does not interfere with results (Figure S6B).

In the case of NRU (Figure S7), we found an increase in absorbance in parallel to the tested concentration of TiO_2_, but this was substantially higher in the case of NPs. One explanation for this phenomenon could be that the solvent used to dissolve the Neutral Red is a mixture of aqueous solutions that favors the precipitation of TiO_2_ NPs. In contrast, in the MTT assay, NPs remain solubilized or in stable clear suspension because the solvent used to dissolve the formazan salt is DMSO, and thus, NPs do not interfere with absorbance determination.

In the case of LDH, no interactions or interferences were found (Figure S8).

According to these observations, the maximum concentration studied when assessing cell viability with MTT and NRU was set at 100 µg/mL, and supernatant was transferred to another plate to avoid reading interferences.

#### 3.4.2. Cytotoxicity of TiO_2_ Particles on HaCaT Cells

**Figure 4:**
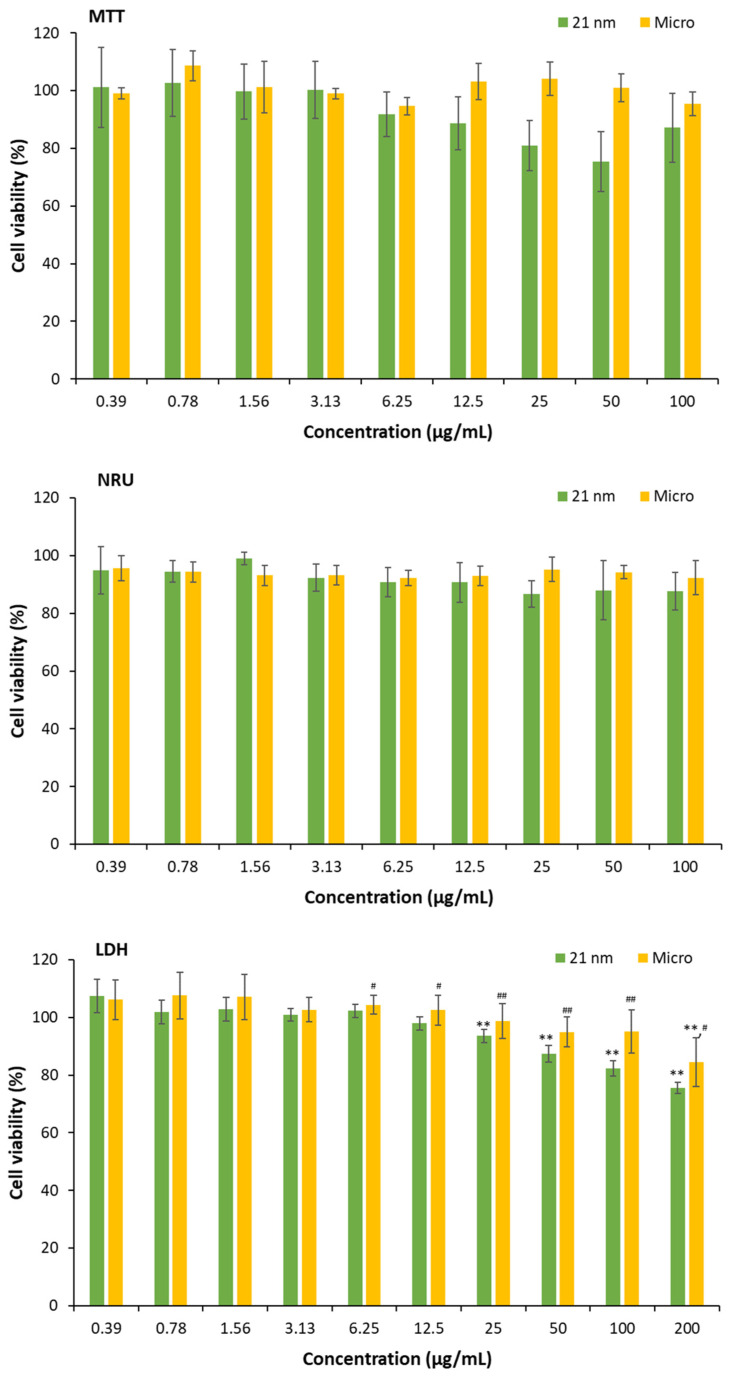
Cell viability obtained by the MTT, NRU and LDH assays after 24 h incubation with 21 nm TiO_2_ or micro-sized particles. Cell viability is expressed as the percentage of viable cells relative to untreated HaCaT cells (100%). Results are expressed as the mean ± SEM of at least three independent assays. Statistical differences were assessed by a one-way ANOVA followed by a Dunnett post hoc test to determine the effect of different concentrations (* *p* < 0.05 and ** *p* < 0.01) or a Student’s *t*-test to assess the effect of particle size (^#^ *p* < 0.05 and ^##^ *p* < 0.01).

The viability of HaCaT cells was studied by MTT, NRU, and LDH after 24 h incubation with TiO_2_ particles (Figure 4). As observed, from 25 µg/mL to higher concentrations of 21 nm TiO_2,_ cell viability is reduced (*p* < 0.01) when assessed by LDH but not with NRU or MTT, with the latter assay presenting high dispersion, although no interferences or interactions were detected at the concentrations assessed. In the case of micrometric particles, no alterations were detected with the MTT or NRU assays, but some very low cytotoxicity was detected with LDH at the maximal concentration assayed (about a 20% decrease in cell viability, *p* < 0.05). Our MTT assay data at 100 μg/mL are similar to those recently obtained with TiO_2_ of 15 and 30 nm by other authors [42], who calculated that the inhibitory concentration was higher than 500 μg/mL.

However, depending on the method used to assess membrane integrity, cytotoxicity may be affected. In other words, the functionality of the lysosomes is unchanged, while the presence of nanoparticles favors the release of the enzyme lactate dehydrogenase (a greater number of living cells and, therefore, a lower percentage of viability).

It has been described in the literature that cells begin to release LDH when exposed to TiO_2_ NPs at 100 μg/mL [43]. Here, we have found that the increase in LDH release starts at 25 μg/mL, reaching 25% cytotoxicity at 100 μg/mL, which is a higher value than that described by these authors [43] but lower than the amount detected by [42]. The adsorption of proteins from the medium, as well as other aspects described elsewhere such as agglomeration or sedimentation, likely alters the particle surface properties, masking reactive surface sites and potentially modulating their biological reactivity to become heterogenous [25]. These effects together may contribute to the attenuated low cytotoxicity observed in the case of MTT and NRU. Whereas MTT and NRU evaluate cytotoxicity exerted on cell metabolism or lysosomal function, which allows particles to enter the cell, LDH detects the breakdown of the plasmatic cellular membrane. For this reason, to avoid an underestimation of cytotoxicity, the use of different in vitro assays is recommended.

#### 3.4.3. Phototoxicity of TiO_2_ Particles on HaCaT Cells

**Figure 5:**
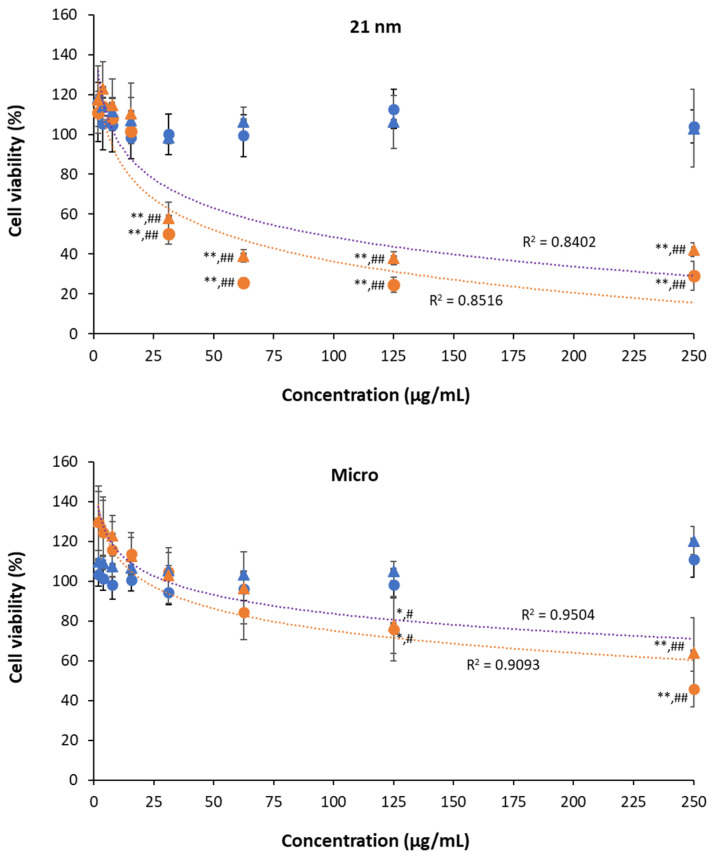
Phototoxicity of nano- and micro-sized TiO_2_ particles evaluated by the MTT (circle) and NRU (triangle) assays after 24 h of being exposed to 4 J/cm^2^ of UVA (orange) or maintained in dark conditions (blue). Cell viability is expressed as the percentage of viable cells relative to untreated HaCaT cells. Results are expressed as the mean ± SEM for at least three independent assays. Statistical differences were assessed by a two-way ANOVA followed by a Dunnett post hoc test to determine the effect of different concentrations (* *p* < 0.05 and ** *p* < 0.01) or Student’s *t*-test to assess the effect of irradiation (^#^ *p* < 0.05 and ^##^ *p* < 0.01). IC_50_ (half-maximal inhibitory concentration) was calculated by the best correlation between independent and dependent variables (R^2^) only in the case of UVA conditions.

Phototoxic behavior was evaluated for both nano- and micro-sized TiO_2_ by the calculation of PIF (Table 4) for each cellular viability method (Figure 5). For this objective, cells treated with micro- and nano-sized TiO_2_ were exposed to UVA light at 4 J/cm^2^. The PIF for nanoparticles was greater than (MTT) or equal (NRU) to 3, demonstrating that TiO_2_ NPs can cause phototoxicity in the HaCaT cell line according to [20]. In contrast, micro-sized TiO_2_ resulted in a PIF of 1.3 in the MTT and NRU assays, showing non-phototoxic potential as expected. Our results agree with [44], where micro-sized TiO_2_ samples did not induce phototoxicity, contrary to TiO_2_ nanoparticles of varying sizes, which exhibited phototoxic effects. Another aspect that accounts for reactivity is the crystal phase of anatase being the most photoreactive [45] and thus avoided in cosmetic formulations or in some cases combined with rutile [46]. However, the observed phototoxicity here for 21 nm TiO_2_ could be attributed to enhanced photocatalytic activity arising from the coexistence of anatase/rutile [33,44] and the small size [33]. The generation of ROS also seems to be correlated with the phototoxic potential depending on the nanoparticle’s size (specific area of particles) and shape [33,44,47], and ROS generation is stimulated via lysosomal membrane permeabilization [48]. Thus, the phototoxic behavior reported here for 21 nm TiO_2_ particles should be mainly attributed to their small size when compared to the micro-sized ones, as anatase is the major phase in the powder. It would be necessary to compare micro-sized TiO_2_ with the same percentage of polymorphic forms to achieve a better evaluation of its potential photoreactivity in the presence of rutile.

### 3.5. Genotoxicity of TiO_2_ Particles on HaCaT Cells

**Figure 6:**
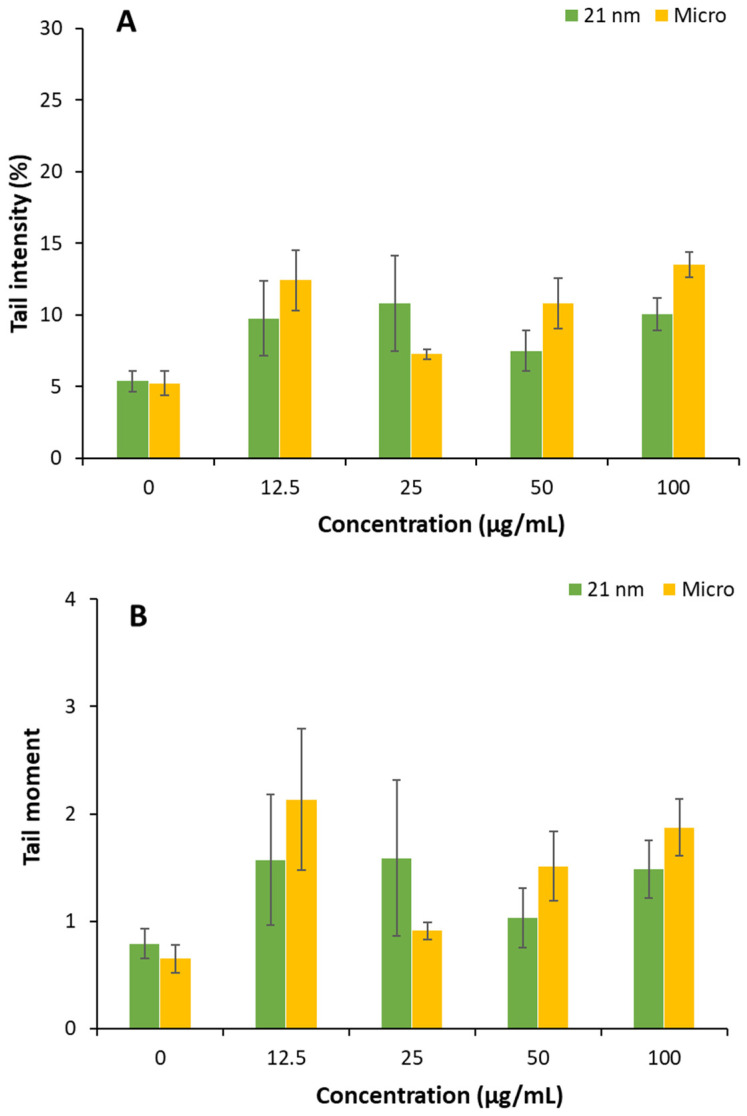
Genotoxicity of nano- and micro-sized TiO_2_ particles in HaCat cells determined by the comet assay during 24 h exposure. Data represents the mean percentage of tail intensity (**A**) and tail moment (**B**) of three independent experiments (50 cells/concentration). Error bars represent standard errors of the mean (SEM). Statistical differences were assessed by a two-way ANOVA followed by Student’s *t*-test. Significant differences between conditions and controls were set at * *p* < 0.05 and ** *p* < 0.01.

The genotoxic potential of TiO_2_ particles was investigated by evaluating DNA strand breaks using the comet assay. After 24 h of cell exposure, the results show that only micro-sized TiO_2_ particles produced significant DNA damage at 12.5, 50, and 100 µg/mL when the damage was measured as the % of DNA in the tail. Notably, a significant increase in DNA damage was also observed in cells exposed to micro-sized TiO_2_ at a 12.5 µg/mL concentration when damage was measured using the tail moment. In contrast, TiO_2_ nanoparticles did not elicit any significant genotoxic effects under the conditions tested (Figure 6).

Numerous studies have investigated the impact of TiO_2_ nanoparticles on various in vitro cell models, yet the findings remain controversial, as reviewed by [49]. The disparity of reported positive and negative results could be attributed to differences in the physicochemical properties of the TiO_2_ particles, including particle size, crystal structure, surface area, and surface charge, as well as variations in experimental protocols such as exposure time, concentration, and cell type [50]. Our results do not indicate genotoxic potential of nano-sized TiO_2_, consistent with [51], who reported similar results using a 25 nm anatase–rutile mixture of TiO_2_ in HaCat cells. In contrast, the observed genotoxicity for TiO_2_ microparticles is likely linked to oxidative stress and mechanical damage associated with cellular uptake. When mammalian cells encounter insoluble particles such as TiO_2_, they may internalize them through phagocytosis and generate ROS, leading to oxidative damage, including lipid peroxidation and DNA strand breaks [52,53]. However, further studies are needed to verify whether oxidative stress is indeed the primary mechanism involved in these genotoxic effects because both particles studied here, nano and micro, form agglomerates under the conditions assessed. Another aspect that needs to be clarified is whether the low percentage of rutile present in 21 TiO_2_ could eventually reduce the genotoxic risk associated with the anatase polymorphism described [50].

## 4. Conclusions

Sunscreen formulations usually contain TiO_2,_ which is regarded as a safe molecule with the capacity to protect against UVA and UVB rays. Currently, formulations commonly use nano-sized instead of micro-sized titanium oxide to avoid aesthetic complaints. However, several studies have questioned the safety of TiO_2_ NPs due to their capacity to promote ROS generation and the potential adverse effects related to them, especially when the anatase phase is the majority. Here, we have compared different toxic properties of 21 nm TiO_2_ particles with micro-sized ones. Our findings indicate that although NPs were more hemolytic than micro-sized TiO2, the hemolysis induced at the highest concentrations studied, difficult to find on blood circulation, is low. Moreover, the effects on the coagulation cascade observed here are independent of particle size. In the case of cytotoxicity and phototoxicity, 21 nm TiO_2_ tends to be more cytotoxic at concentrations equal to or higher than 25 µg/mL, but attention should be paid to the capacity to induce phototoxic reactions, and this aspect needs further investigation, specifically to understand the inclusion of a low percentage of crystals. Finally, our results do not indicate a genotoxic potential associated with 21 nm TiO_2_. We concluded that 21 nm TiO_2_ shows slightly cytotoxic behavior, with its potential phototoxicity being the aspect that should be studied in depth to confirm consumer safety.

## Figures and Tables

**Table 1:** Characteristics of the commercial TiO_2_ particles studied.

Reference	Particle Size	Form	Crystal Structure	Purity	Surface Area
718467	21 nm (TEM)	White powder	80% anatase 20% rutile	≥99.5%	35–65 m^2^/g
14027	n. s.	White powder	n. s.	99.0–100.5%	n. s.

n. s.—not supplied.

**Table 2:** TiO_2_ crystalline phases and crystal size in nanometers (nm) obtained by XRD analysis.

Polymorph	21 nm	Micro
Anatase *	86.4%	100%
	18 nm	123 nm
Rutile ^#^	13.6%	n. d.
	31 nm	n. d.

* PDF 0-21-1272; ^#^ PDF#0-21-1276 size; n. d.: not detected.

**Table 3:** Hydrodynamic diameter (nm) of TiO_2_ particles after incubation in different media for 2 h and 24 h.

Particle Size	Incubation Time	PBS *	PBS + BSA *	PBS + Fib *	DMEM *
21 nm	2 h	458.0 ± 6.0	362.8 ± 16.4	403.5 ± 33.8	311.4 ± 7.2 ^$^
	24 h	485.4 ± 15.1	348.1 ± 6.6 ^$$^	404.7 ± 57.1	288.2 ± 17.2 ^$$^
Micro	2 h	478.5 ± 20.5	236.3 ± 20.6 ^$$^	402.5 ± 16.18	280.6 ± 12.4 ^¥¥^
	24 h	606.3 ± 11.4 ^¥¥^	158.8 ± 10.9 ^$$, ¥¥^	428.4 ± 5.1 ^$$^	280.0 ± 2.9 ^$$^

* Results are expressed as the mean ± standard error of the mean (SEM) of 3 independent experiments. Nanoparticles (1.0 mg/mL) were incubated in phosphate-buffered saline (PBS, pH 7.4), in PBS containing 2 mg/mL of albumin (PBS + BSA) or fibrinogen (PBS + Fib), or in DMEM with 5% FBS for 2 and 24 h at 37 °C. To compare the effect of media on hydrodynamic diameter, a two-way ANOVA analysis was performed followed by a Bonferroni post hoc test to compare with PBS (^$^ *p* < 0.05 and ^$$^ *p* < 0.01), and Student’s *t*-test was used to evaluate the effect of time in each condition (^¥^ *p* < 0.05 and ^¥¥^ *p* < 0.01).

**Table 4:** Photo-irritation factor (PIF *) obtained for TiO_2_ particles by MTT and NRU assays.

Particle Size	MTT	NRU
21 nm	4.6 ± 0.5	3.0 ± 0.5
Micro	1.3 ± 0.3	1.3 ± 0.3

* Results are expressed as the mean ± standard error of the mean (SEM) of at least 3 independent experiments.

## Data Availability

Data are contained within the article and Supplementary Materials.

## Supplementary Materials

The following supporting information can be downloaded at https://www.mdpi.com/article/10.3390/nano15120951/s1. Figure S1: Size distribution of 21 nm (A) and micro-sized (B) TiO_2_ particles; Figure S2: X-ray powder diffraction diagram of 21 nm (A) and micro-sized (B) TiO_2_ powder samples with patterns of the identified phases superimposed (blue: anatase; green: rutile); Figure S3. Zeta potential distribution curve for 21 nm TiO_2_ particles suspended in distilled water (0.1 mg/mL); Figure S4. Zeta potential distribution curve for micro-sized TiO_2_ particles suspended in distilled water (0.1 mg/mL); Figure S5: Hemolytic activity observed after 24 h of incubation with 1 mg/mL of TiO_2_ 21 nm (A) and microparticles (B) at room temperature in dark conditions; Figure S6. Interferences of nano- and micro-sized TiO_2_ with MTT assay; Figure S7. Interferences of nano- and micro-sized TiO_2_ with NRU assay; Figure S8. Interferences of nano- and micro-sized TiO2 with LDH assay.

## Abbreviations

The following abbreviations are used in this manuscript:

aPTT	Activated Partial Thromboplastin Time
CCiTUB	Centres Científics i Tecnològics de la Universitat de Barcelona
DLS	Dynamic Light Scattering
DMSO	Dimethyl Sulfoxide
FBS	Fetal Bovine Serum
HD	Hydrodynamic Diameter
LDH	Lactate Dehydrogenase
MTT	Thiazolyl Blue Tetrazolium Bromide
NPs	Nanoparticles
NR	Neutral Red Solution
NRU	Neutral Red Uptake
PBS	Phosphate-Buffered Saline Solution
PDI	Polydispersity Index
PIF	Photo-Irritation Factor
PT	Prothrombin Time
RBC	Red Blood Cell
ROS	Reactive Oxygen Species
TEM	Transmission Electron Microscopy
UV	Ultraviolet
