# Supplementary material for: Optimizing Sunscreen Safety: The Impact of TiO2 Particle Size on Toxicity and Biocompatibility
Source: Nanomaterials (Basel). 2025 Jun 19;15(12):951. doi: 10.3390/nano15120951 (PMC12196315; doi:10.3390/nano15120951)
Supplement: Supplementary file 1 [file nanomaterials-15-00951-s001.zip › nanomaterials-3634876-supplementary.pdf]

Supplementary Materials

Optimizing Sunscreen Safety: The Impact of TiO_2 Particle Size on Toxicity and Biocompatibility

Adriana S. Maddaleno [1,2], Clàudia Casellas[3], Elisabet Teixidó[3], Laia Guardia-Escote[3], M. Pilar Vinardell [1,2*] and Montserrat Mitjans [1,2*]

[1] Physiology, Dpt. Biochemistry and Physiology, Universitat de Barcelona. adrianamaddaleno@ub.edu; laura.marfa.89@gmail.com; mpvinardellmh@ub.edu
[2] Institute of Nanoscience and Nanotechnology, Universitat de Barcelona
[3] Toxicology, Dpt. Pharmacology, Toxicology and Therapeutic Chemistry, Universitat de Barcelona. Clcasell8@alumnes.ub.edu; eteixido1511@ub.edu; laia.guardia@ub.edu
* Correspondence: mpvinardellmh@ub.edu; montsemitjans@ub.edu;

1.- Transmission electronic microscopy (TEM).

From the microphotographs taken by the JEOL JEM LaB6-2100f microscope and the use of DigitalMicrograph® (Gatan Microscopy Suit) we have measured the diameter of the particles studied). Size distribution of the particles for each TiO2 powder is presented in Figure S1.

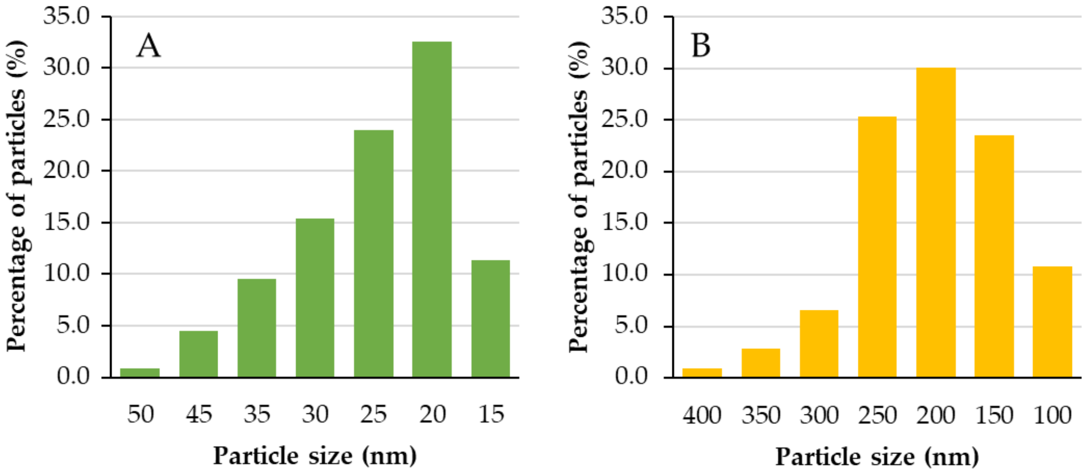

Figure S1. Size distribution of 21 nm (A) and micro-sized (B) TiO_2 particles. From a set of 15 microphotographs diameters of 100 different particles were measured. Mean diameter for 21 nm obtained was 22.1 ± 0.5 and 177.6 ± 4.3 for micro-sized TiO_2 particles.

2.- X-ray diffraction (XRD).

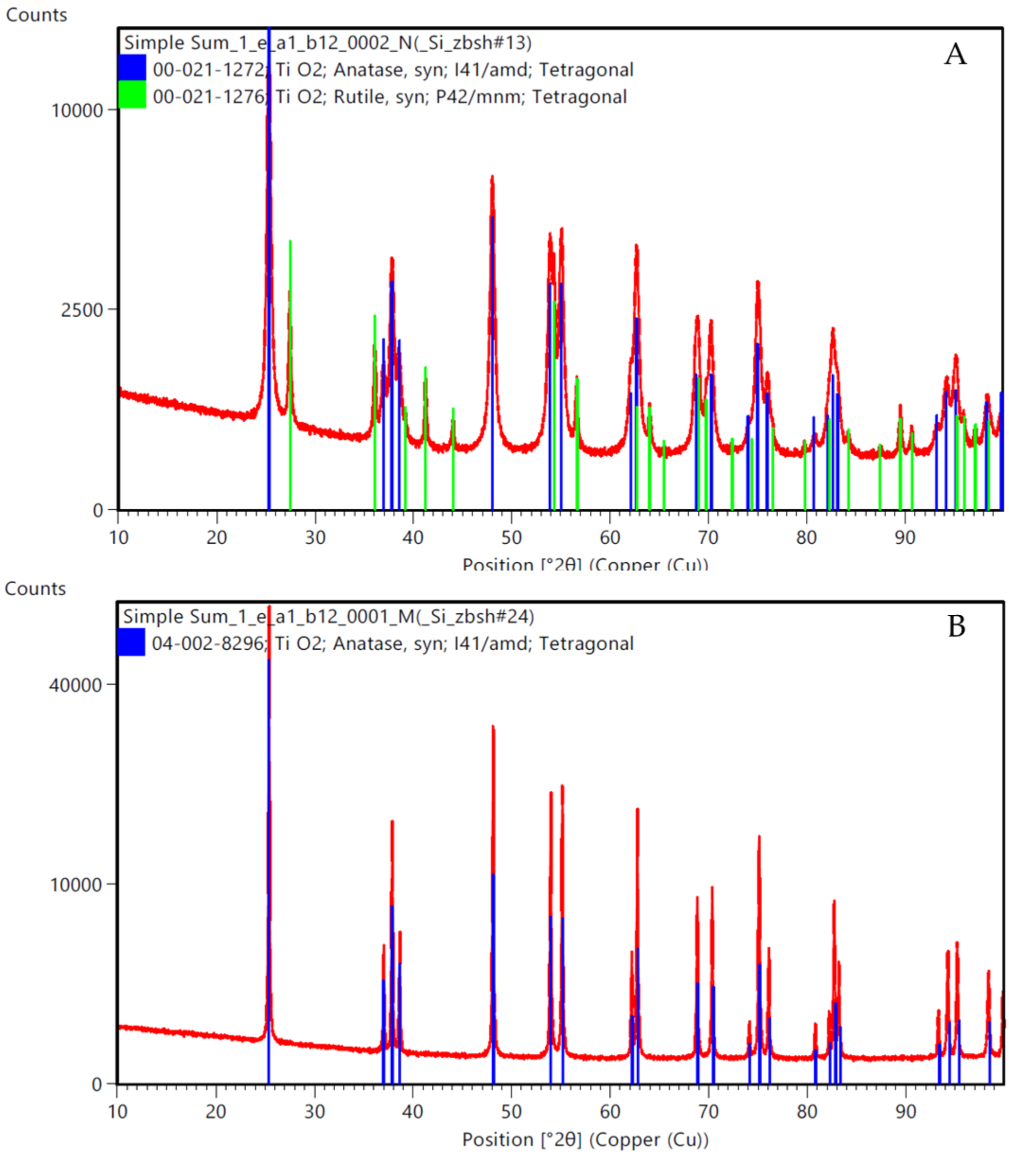

Figure S2. X-ray powder diffraction diagram of 21 nm (A) and micro-sized (B) TiO_2 powder sample with patterns of the identified phases superimposed (blue: anatase; green: rutile).

<u>3.- Zeta potential measurements.</u>

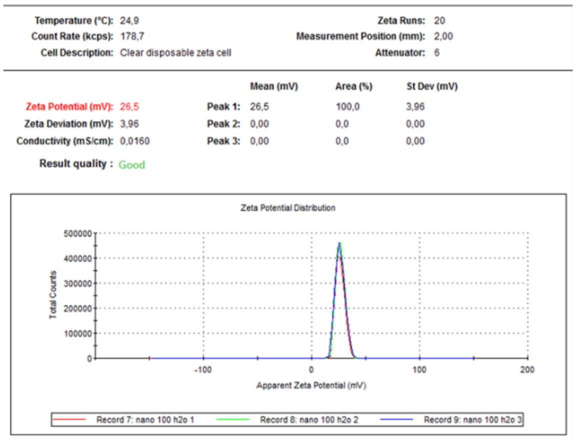

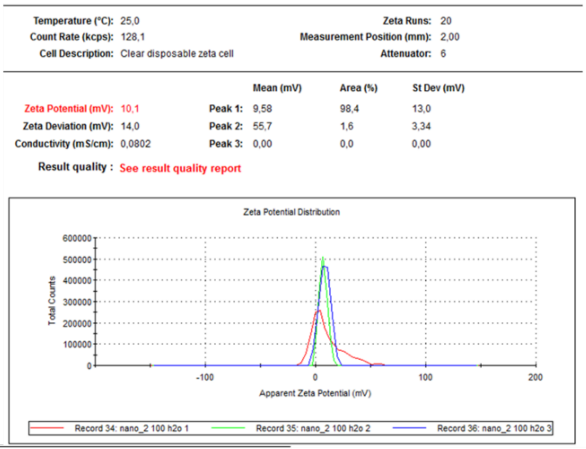

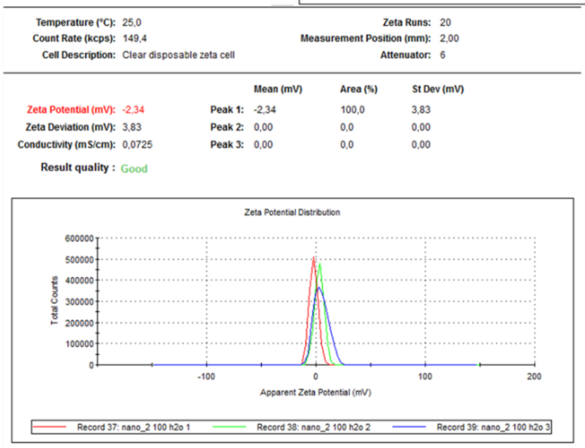

Figure S3. Zeta potential distribution curve for 21 nm TiO_2 particles suspended in distilled water (0.1 mg/mL). All measurements were conducted at 25 °C using disposable folded capillary cells. Each sample was measured almost three times, and the average zeta potential value along with standard deviation was reported to assess colloidal stability.

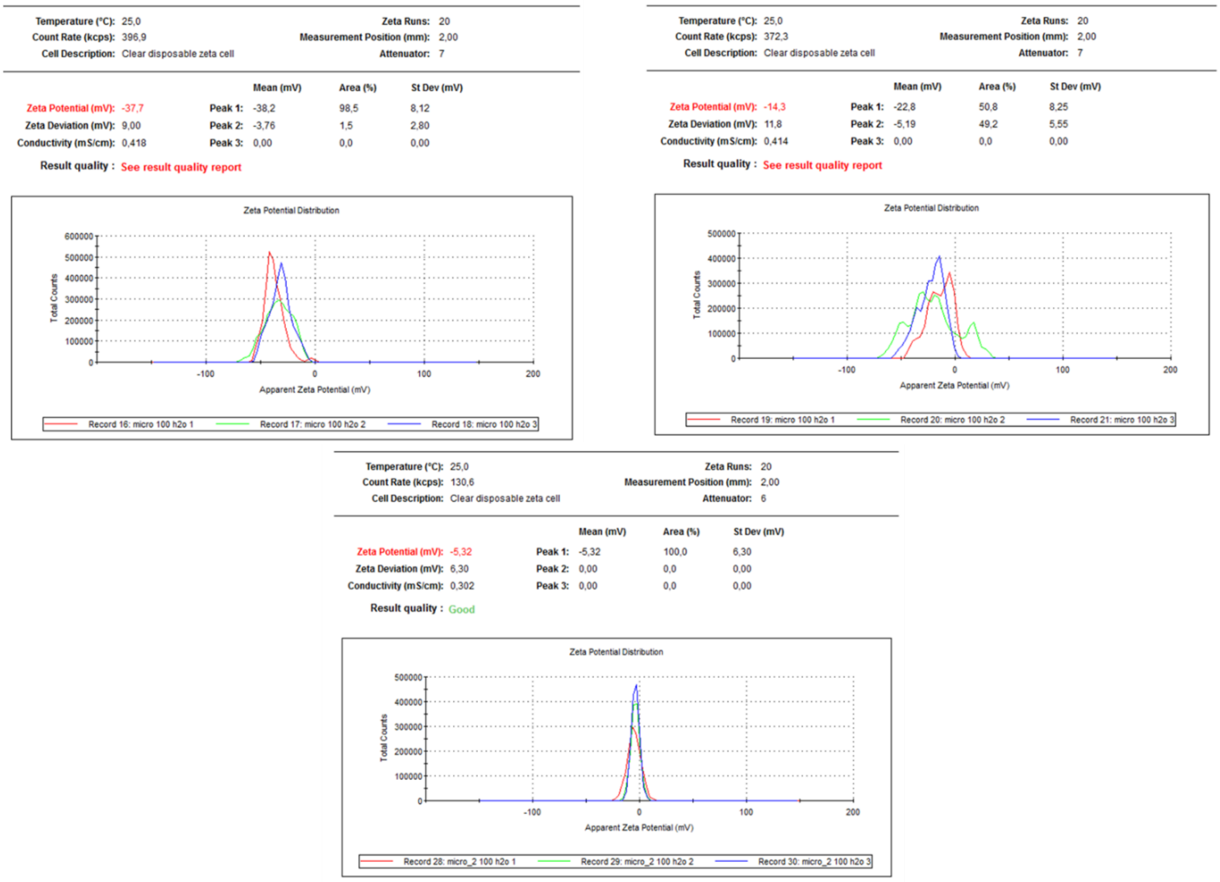

Figure S4. Zeta potential distribution curve for micro-sized TiO_2 particles suspended in distilled water (0.1 mg/mL). All measurements were conducted at 25 °C using disposable folded capillary cells. Each sample was measured almost three times, and the average zeta potential value along with standard deviation was reported to assess colloidal stability.

4.- Study of hemolytic activity induced by TiO_2 after 24 hours of incubation at room temperature.

In Figure S1 is shown the maximal hemolysis achieved after incubating an aliquot of 25 µL red blood cells suspension with 1 mg/mL nano- (A) or micro-sized (B) TiO_2. No hemolytic effect is observed in the case of micro-sized TiO_2 while for nano-sized less than 30% hemolysis is recorded.

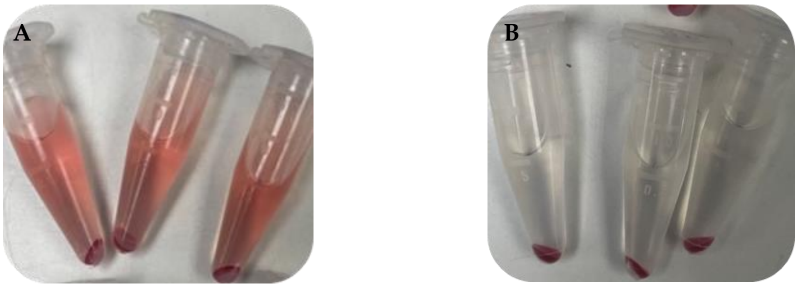

Figure S5. Hemolytic activity observed after 24 hours of incubation with 1 mg/mL of TiO_2 21 nm (A) and microparticles (B) at room temperature in dark conditions.

5.- Study of potential interactions or interferences of TiO_2 NPs with cytotoxic assays

The potential interferences of TiO_2 with reagents used in MTT, NRU and LDH methods as well as potential interferences in readings have been studied in acellular conditions (500 to 1.95 μg/mL).

Figure S6 shows the results obtained in the case of MTT. Absorbances obtained for each concentration (Figure S6A) in acellular conditions were compared with absorbance obtained for untreated cells showing that there exists some interference independent on the concentration assayed. However, when background was subtracted and at the percentage of interference calculated respect to maximal absorbance expected (viable cells) less than 10% (6,1%) was found at the maximal concentration tested (500 μg/mL) for nano-sized TiO_2 (Figure S6B) and being negligible at the rest of concentrations studied. No interferences were recorded for micro-sized particles.

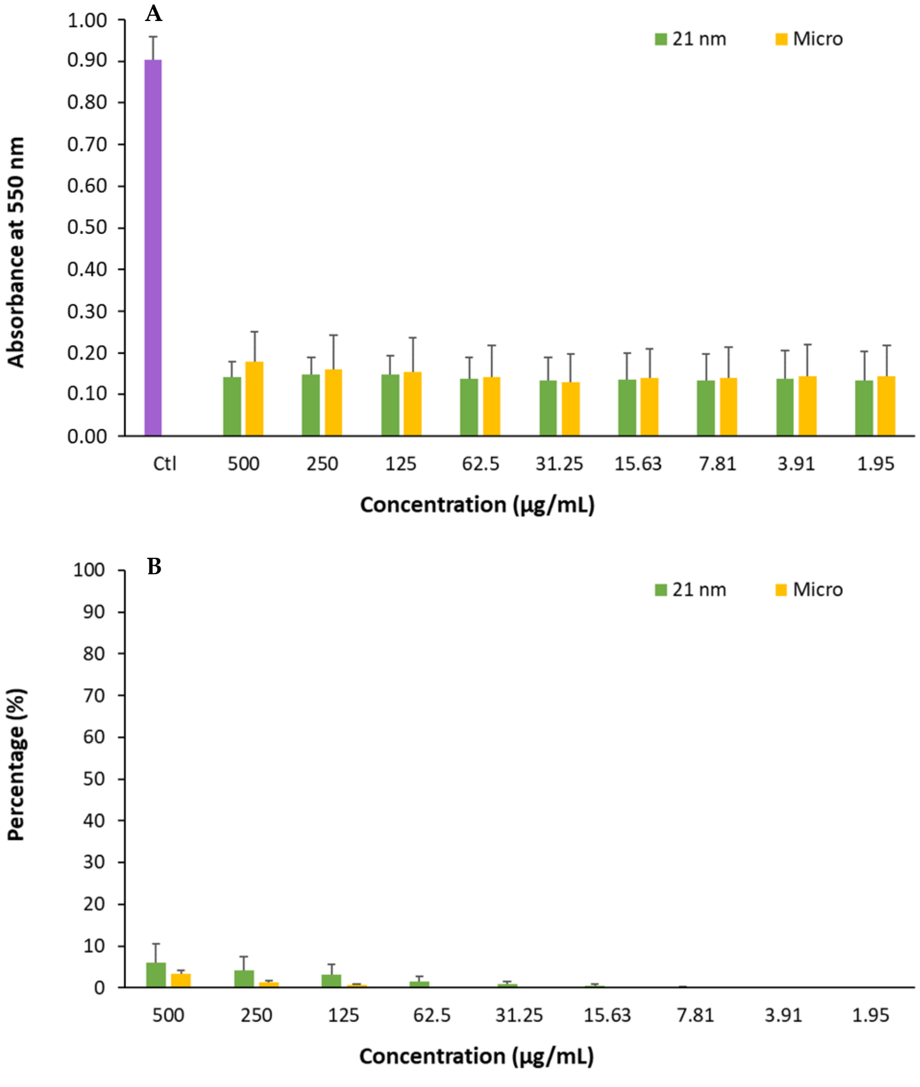

Figure S6. Interferences of nano- and micro-sized TiO_2 with MTT assay. Results are expressed as mean ± standard deviation of at least 3 independent assays. A: absorbance; B: percentage of absorbance respect to absorbance values of control cells (untreated) as a measure of maximal viability expected. Values of control cells were obtained from historical data of at least 7 experiments.

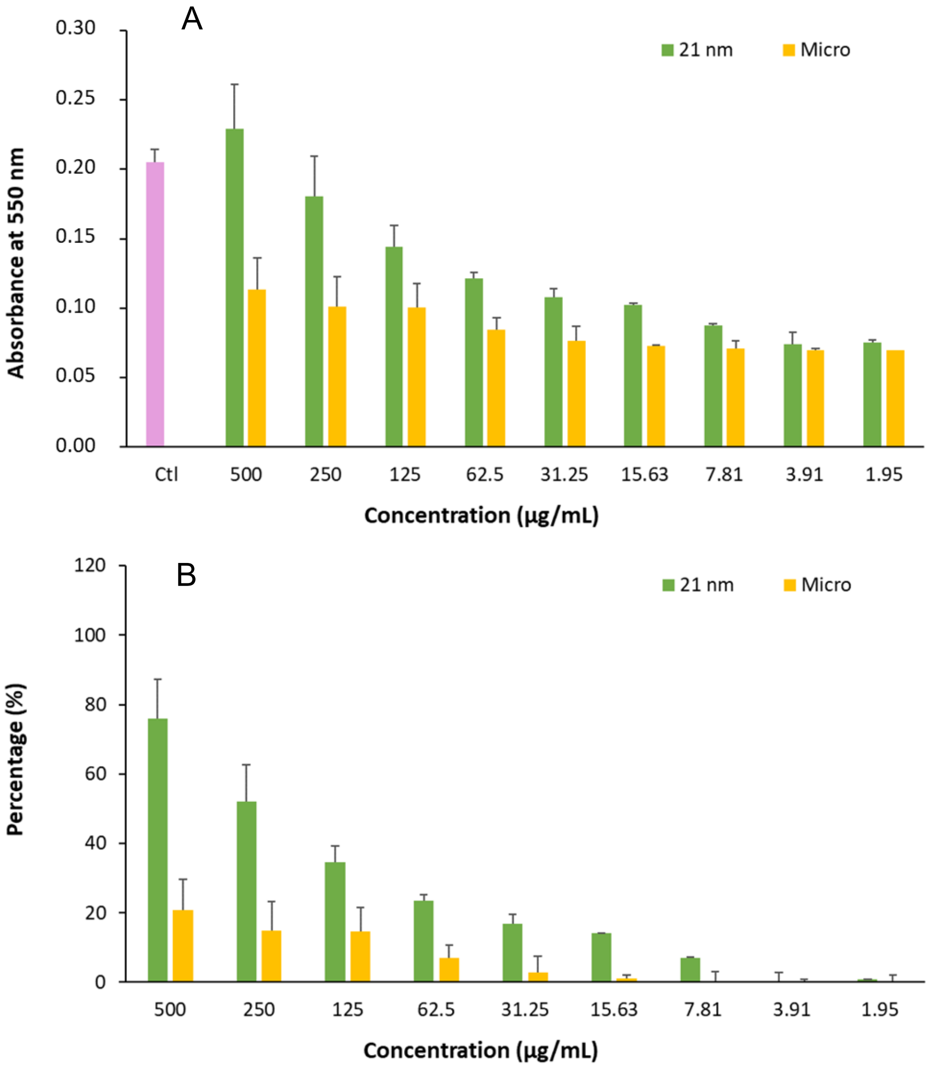

Figure S7. Interferences of nano- and micro-sized TiO_2 with NRU assay. Results are expressed as mean ± standard deviation of at least 3 independent assays. A: absorbance; B: percentage of absorbance respect to absorbance values of control cells (untreated) as a measure of maximal viability expected. Values of control cells were obtained from historical data of at least 7 experiments.

Finally, in the case of LDH (Figure S4A and B) no interferences were found.

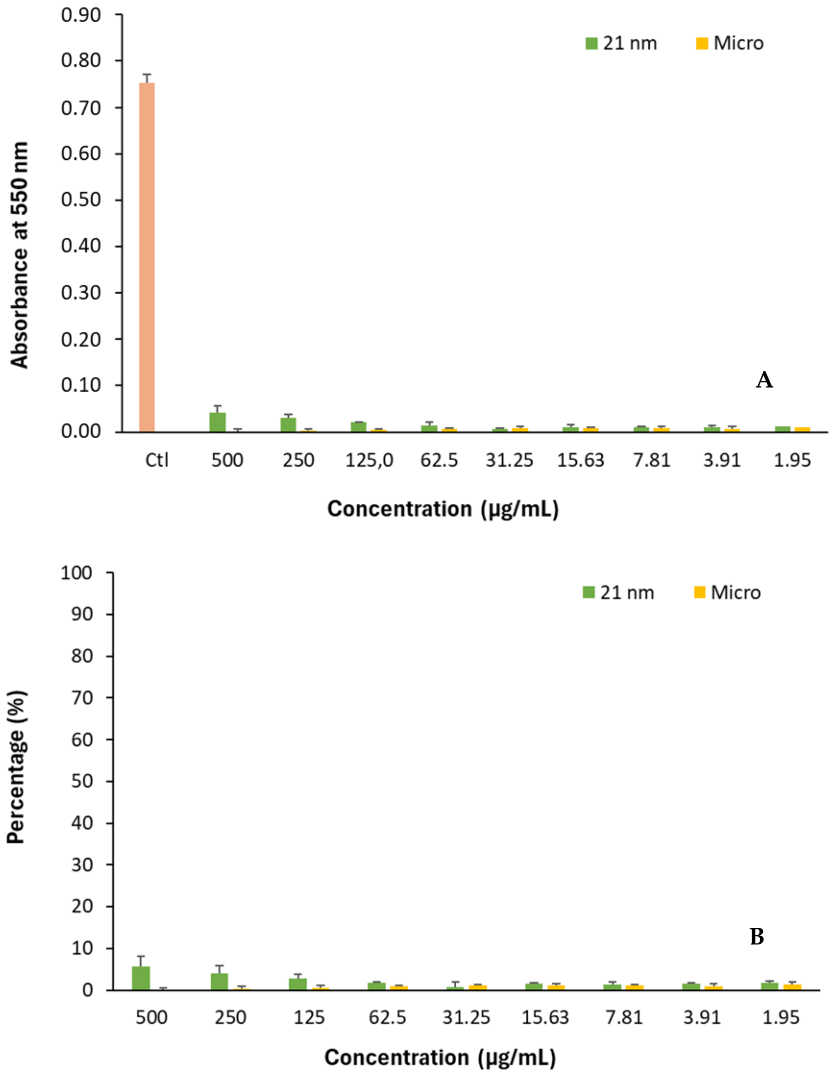

Figure S8. Interferences of nano- and micro-sized TiO_2 with LDH assay. Results are expressed as mean ± standard deviation of at least 2 independent essays. A: absorbance; B: percentage of absorbance respect to cells treated with TritonX-100 as controls of maximal cytotoxicity expected. Values of control cells were obtained from historical data of at least 7 experiments.
